# One-Step Nucleic Acid Amplification (OSNA): A fast molecular test based on CK19 mRNA concentration for assessment of lymph-nodes metastases in early stage endometrial cancer

**DOI:** 10.1371/journal.pone.0195877

**Published:** 2018-04-26

**Authors:** Francesco Fanfani, Giorgia Monterossi, Viola Ghizzoni, Esther D. Rossi, Giorgia Dinoi, Frediano Inzani, Anna Fagotti, Salvatore Gueli Alletti, Francesca Scarpellini, Camilla Nero, Angela Santoro, Giovanni Scambia, Gian F. Zannoni

**Affiliations:** 1 Department of Medicine and Aging Sciences, University "G. D’Annunzio" of Chieti-Pescara, Chieti, Italy; 2 Division of Gynecologic Oncology, Department of Women and Child Health, Catholic University of the Sacred Heart, Rome, Italy; 3 Gynecologic Oncology Pathology Unit, Department of Women and Child Health, Catholic University of the Sacred Heart, Rome, Italy; Universita degli Studi di Napoli Federico II, ITALY

## Abstract

**Introduction:**

The aim of the current study is to evaluate the detection rate of micro- and macro-metastases of the One-Step Nucleic Acid Amplification (OSNA) compared to frozen section examination and subsequent ultra-staging examination in early stage endometrial cancer (EC).

**Material and methods:**

From March 2016 to June 2016, data of 40 consecutive FIGO stage I EC patients were prospectively collected in an electronic database. The sentinel lymph node mapping was performed in all patients. All mapped nodes were removed and processed. Sentinel lymph nodes were sectioned and alternate sections were respectively examined by OSNA and by frozen section analysis. After frozen section, the residual tissue from each block was processed with step-level sections (each step at 200 micron) including H&E and IHC slides.

**Results:**

Sentinel lymph nodes mapping was successful in 29 patients (72.5%). In the remaining 11 patients (27.5%), a systematic pelvic lymphadenectomy was performed. OSNA assay sensitivity and specificity were 87.5% and 100% respectively. Positive and negative predictive values were 100% and 99% respectively, with a diagnostic accuracy of 99%. As far as frozen section examination and subsequent ultra-staging analysis was concerned, we reported sensitivity and specificity of 50% and 94.4% respectively; positive and negative predictive values were 14.3% and 99%, respectively, with an accuracy of 93.6%. In one patient, despite negative OSNA and frozen section analysis of the sentinel node, a macro-metastasis in 1 non-sentinel node was found.

**Conclusions:**

The combination of OSNA procedure with the sentinel lymph node mapping could represent an efficient intra-operative tool for the selection of early-stage EC patients to be submitted to systematic lymphadenectomy.

## Introduction

Endometrial cancer (EC) is the sixth most common gynecologic malignancy in women worldwide with around 300,000 new cases reported annually [1]. The prognostic value of lymphadenectomy for patients with early stages is a matter of debate. Despite this fact, presence of nodal metastasis is an important element to determine the appropriate adjuvant management. Studies on the therapeutic effect of systematic lymphadenectomy showed contradicting results [2,3]. In particular, two randomized studies and one meta-analysis showed that pelvic lymphadenectomy had no impact on the survival of patients with early-stage endometrial cancer with increased morbidity rates [2–4]. In contrast, retrospective data suggest that patients who underwent systematic lymphadenectomy (LND) improved survival over those who had limited or no sampling performed [5].

With the aim to reduce surgical morbidity and to improve the detection of nodal metastases, sentinel lymph node (SLN) mapping has been introduced in the management of these patients showing convincing results [6–8]. Since 2015, the National Comprehensive Cancer Network (NCCN) guidelines proposed to perform the SLN mapping in selected early stage EC patients [9].

The recent introduction of green indocyanine (ICG) as a lymphatic tracer improves the success of SLN identification [10–11], so that ultra-staging based on multiple H&E sections combined with immunohistochemistry (IHC), added to conventional routinary histology, increases the likelihood of identifying micro-metastases in approximately 15–20% of the cases [9]. As reported by some authors [12], the SLN ultra-staging protocol consists in cutting two adjacent 5-μm sections at each of two levels, 50-μm apart, from each paraffin block lacking metastatic carcinoma on routinely haematoxylin and eosin (H&E) staining. At each level, one slide is stained with H&E and the other one with IHC using the anti-cytokeratin AE1:AE3 (Ventana Medical Systems, Inc., Tucson, AZ) for a total of five slides per block in order to detect a low-volume metastatic disease. Low-volume metastatic disease, as defined in the breast cancer literature, includes isolated tumor cells (ITCs) and micro-metastases (MMs). ITCs are defined as microscopic clusters and single cells measuring ≤ 0.2 mm, and MMs as a focus of metastatic tumor cells measuring >0.2 mm and ≤2 mm [13].

Although the use of extensive histology and IHC have improved the detection rate of LN metastases, these methods are burdensome, time consuming and not suited for rapid, intraoperative diagnoses, so that patients could be considered for systematic lymphadenectomy and adjuvant therapy only in the post-surgical evaluation. To overcome this limit, Sysmex Corporation (Kobe, Japan) has developed an innovative molecular method, the One Step Nucleic Acid Amplification (OSNA) reaction, that in combination with the reagent “Lynoamp BC”, allows the rapid and accurate detection of cytokeratin 19 (CK19) mRNA in metastatic lymph nodes of breast, gastric and colon cancer patients [14–16].

OSNA assay consists in the homogenization o of a lymph node followed by reverse-transcription loop-mediated isothermal amplification (RT-LAMP) of a target mRNA [17]. The OSNA method is characterized by a quantitative measurement of the target mRNA in a metastatic lymph node, a brief reaction time for the entire process, a high specificity for the target mRNA and an absence of genomic DNA amplification. Previous studies selected CK19 as a single marker target mRNA [14; 18]. CK19 is considered a promising marker with high sensitivity for detecting tumour cells in several solid tumors, including EC [14, 19]. More recently, Lopez-Ruiz et al. [20] confirmed OSNA utility as an incoming tool for the detection of SLN metastases.

Finally, Sysmex Corporation has now developed a new Gene Amplification Detector (RD-210) and a new reagent (LS60R) for the OSNA assay. By using this new system, it will be possible not only to detect lymph node metastasis, but also to gain quantitative information about the size of the metastasis in the lymph node.

The principal aim of the current study is to confirm these data in a consecutive series analyzing the micro- and macro-metastases detection rate of OSNA assay compared to frozen section examination and subsequent ultra-staging analysis.

## Materials and methods

This study involves 40 consecutive FIGO stage I endometrial cancer patients treated between March 2016 and June 2016 at the Division of Gynecologic Oncology of the Catholic University of the Sacred Heart of Rome.

The Department of Women and Child Health of the Catholic University approved this study on January 2016. All patients were informed about the study purposes and signed the informed consent.

Baseline characteristics and surgical and pathological features were recorded for each patient. Histological type was classified according to the 2014 World Health Organization classification of tumors of the endometrium [21].

Surgical staging was performed by minimally invasive approach, and SLN mapping was performed in all patients by ICG injection. Technique and method for SLN mapping was the same in all cases. Retroperitoneal dissection was always the first surgical step. All mapped lymph nodes were removed and carried in dry containers to the pathology laboratory for the OSNA analysis within 30 minutes. According to internal protocol, a systematic pelvic lymphadenectomy was always performed in case of poor differentiated, and/or myometrial invasion more than 50%. Considering each emi-pelvis, when sentinel node/s were not identified, a systematic pelvic lymphadenectomy was performed, and all nodes were evaluated by OSNA analysis. Systematic pelvic and aortic was performed in patients with positive pelvic node or high-risk endometrial cancer patients.

**Fig 1:**
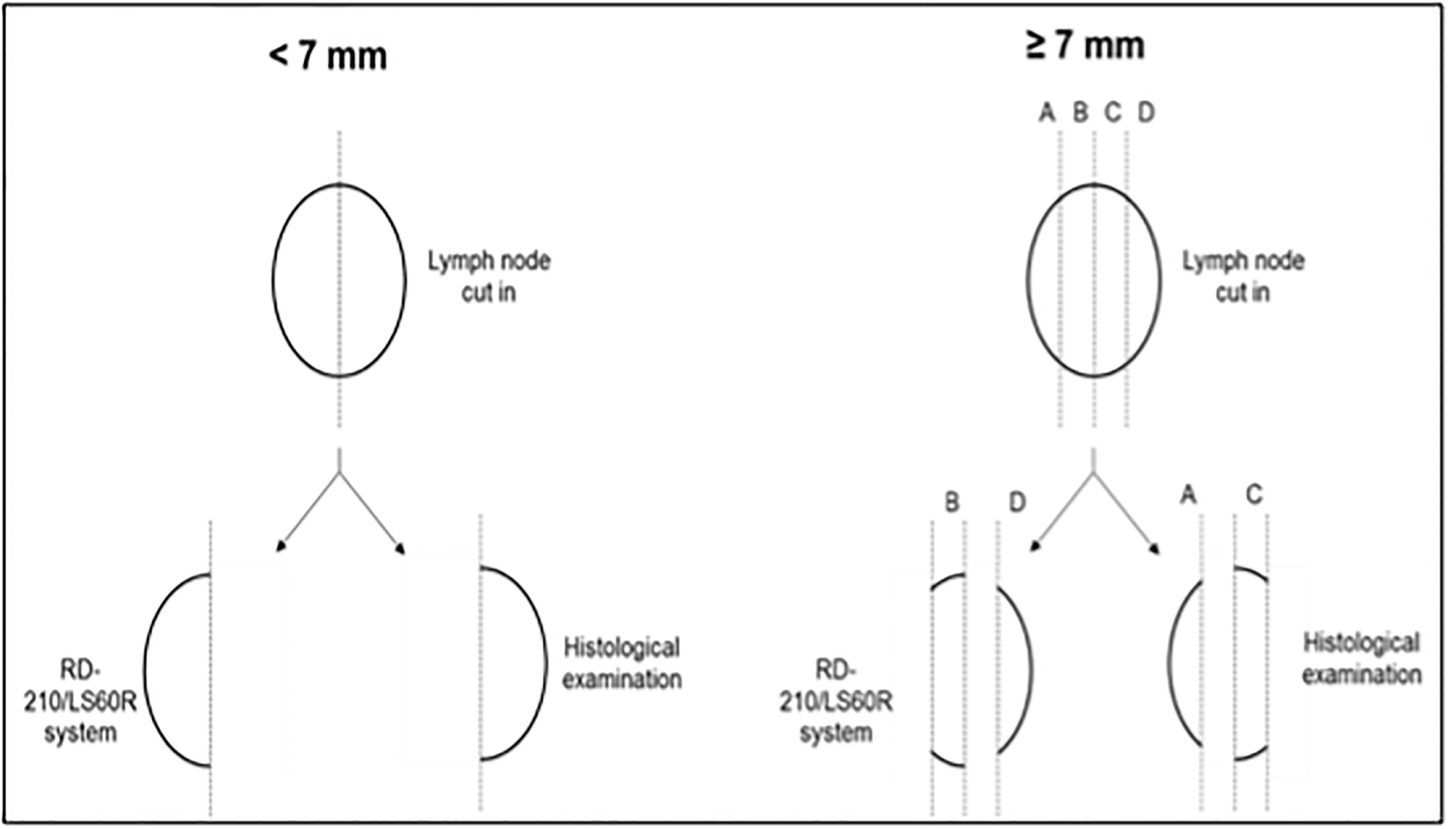
Schematic representation of lymph node processing.

All the samples with the SLN sections for OSNA were defatted, weighed and processed. Size and weight of all removed nodes are evaluated. LNs up to 7 mm thickness are cut into halves with one half attributed to each method, while LNs larger than 7 mm thickness and weighting between 50 mg and 1 g are sectioned into four slices (A, B, C and D) of 1–2 mm in thickness. LN sections B and D are analyzed by the OSNA (RD-210/LS60R) system, compared to slices A and C by frozen section histology (Fig 1).

Large SLNs exceeding 1 g in weight were bilobed and processed as two separate nodes through OSNA method. The smallest SLNs (<0.05 g, as recommended by the manufacturer) precluded the assessment of concordance between the two techniques. Such nodes were however included in the final analysis of the rates of SLN positivity.

### Study procedures

#### Analysis of LNs by OSNA (RD-210/LS60R) system

The OSNA machine processes the whole LN lysate. LN sections are homogenized in 4 ml of lysing buffer for 90 s at 25,000 rpm using stainless steel blades or for 60 s at 10,000 rpm using LYNOPREP blade set and centrifuged for one minute at 10,000 x g. Subsequently, CK19 mRNA is amplified by RT-LAMP in the RD-210 [22]. Automated amplification with a ready-to-use reagent kit (formulated without the necessity of calculation, dilution or pipetting) is performed directly from the sample lysate, with no RNA purification necessary, according to the manufacturer’s instructions. Based on previous studies in breast, gastric and colorectal cancer patients, LNs are defined as ‘negative’ or ‘positive’ according to established cut-off values [14–19]. Therefore, negative nodes are classified for CK 19 mRNA ccP/μl as less than 250. Isolated tumor cells in LNs were reported as a ‘late’ rise i.e. copy numbers rose to < 250 copies/ml. LNs positive for micro-metastases (+) show mRNA CK19 levels of 250–5000 ccP/μl and those nodes with macro-metastases (++) show more than 5,000 mRNA ccP/μl. Leftover lysates are used for RNA extraction, determination of RNA integrity and other molecular techniques.

#### RNA purification and calculation of RNA Integrity Number (RIN)

Total RNA is purified from 150 μl lysate by using RNeasy Plus Mini Kit (QIAGEN) and eluted with 50 μl of RNase-free water automatically by QIAcube (QIAGEN) following manufacturer’s instructions. A LN lysate that was shown to contain intact total RNA in a previous experiment serves as positive control for the RNA purification procedure. The purity of the total RNA is determined by the RIN value which is calculated automatically by Agilent 2100 bioanalyzer (Agilent Technologies). Each sample is measured once. The RNA integrity is considered to be sufficient for molecular techniques, if the RIN value ≥ 5.0.

#### Histopathology of LNs

After frozen section, the residual LN tissue from each block was processed with step-level sections (each step at 200 micron) including haematoxylin and eosin (H&E) and IHC slides.

Established methods of histology are employed on allocated sections comprising H&E. Tissue was cut in 4 mm slices and dried at 60°C. Paraffin is separated using xylol and ethanol (70%-100%).

Our institutional ultra-staging protocol consisted in cutting two adjacent 5-μm sections at each of five levels, 200-μm apart, from each paraffin block lacking metastatic carcinoma on routinary H&E staining. At each level, one slide is stained with H&E and the other one with IHC using the anti-cytokeratin AE1:AE3 (Ventana Medical Systems, Inc., Tucson, AZ) in order to detect a low-volume metastatic disease at a light microscope.

The positivity of LNs is classified according to the size of the tumor deposit as follows: macro-metastases (>2.0 mm); micro-metastasis (>0.2–2.0 mm); and isolated tumor cells (up to 0.2 mm). The histological staging was N1 for macro-metastases, N1mi for micro-metastasis and N0 for isolated tumor cells.

### Statistical analysis

No formal sample size was estimated. The data are described by medians and range or counts and percentages, as suitable. The statistical analysis was carried out with SPSS version 18.0 for Windows (SPSS). Calculations of sensitivity and specificity were performed in order to delineate concordance of OSNA with histopathology.

## Results

Demographics, clinical and histopathological characteristics are summarized in Table 1. The median age at diagnosis was 60 years (range 35–87 years). Final histological diagnosis, based on hysterectomy specimens, included 33 endometrioid (82.5%), 4 serous (10%), 2 clear cells carcinoma (5%), and 1 undifferentiated tumor (2.5%). Tumor grade was G1 in 10 patients (25%), G2 in 15 patients (37.5%), and G3 in 15 (37.5%) patients. Lymph-vascular space involvement was negative in 28 cases (70%) and positive in the remaining 12 cases (30%).

**Table 1:** Demographics and baseline characteristics.

**Patients (n = 40)**	
**Age at diagnosis (range)**	60 (35–87)
**Histology**	
Endometrioid	33 (82.5%)
Serous	4 (10%)
Clear cells	2 (5%)
Anapastic	1 (2.5%)
**Grading**	
G1	10 (25%)
G2	15 (37.5%)
G3	15 (37.5%)
**LVSI**	
Positive	28 (70%)
Negative	12 (30%)
**SLN mapping**	
No	11 (27.5%)
Yes	29 (72.5%)
– Monolateral	– 8 (20.0%)
– Bilateral	– 21 (52.5%)

(LVSI: Lymph-vascular space involvement; SLN: sentinel node)

SLN mapping was efficacious in 29 out of 40 patients (72.5%). In particular, we mapped and removed bilateral and unilateral SLNs in 21 (52.5%) and in 8 (20.0%) patients, respectively. We analyzed a median of 2 SLNs (range 1–4) for patient.

Summary of clinical and histopathological characteristics is resumed in Table 2.

**Table 2:** Summary of clinical and histopathological characteristics.

Case #	Age	SLN mapping	Pe LFN	Ao LFN	OSNA SLN	FS SLN	OSNA LFN	FS LFN	Histotype	Grade	LVSI
1	74	Yes	No	No	2-	2-	No	No	Endometrioid	G2	Yes
2	60	Yes	No	No	2-	2-	No	No	Endometrioid	G2	No
3	60	Yes	No	No	2-	2-	No	No	Endometrioid	G1	No
4	65	Yes	No	No	2-	2-	No	No	Endometrioid	G1	No
5	54	Yes	No	No	2-	2-	No	No	Endometrioid	G2	No
6	69	No	Yes	No	No	No	21-	21-	Endometrioid	G3	Yes
7	75	Yes	No	No	1+, 1-	2-	No	No	Endometrioid	G3	Yes
8	80	Yes	No	No	1+, 1-	2-	No	No	Undifferentiated	G3	No
9	58	No	Yes	No	No	No	2-	2-	Endometrioid	G2	No
10	55	Yes	No	No	2-	2-	No	No	Endometrioid	G1	No
11	57	Yes	Yes	No	1-	1-	No	No	Endometrioid	G2	No
12	53	Yes	No	No	1-	2-	No	No	Endometrioid	G1	No
13	57	Yes	Yes	No	1-	1-	No	No	Endometrioid	G2	No
14	67	Yes	No	No	1-	1-	No	No	Endometrioid	G1	No
15	50	Yes	Yes	No	2-	2-	No	No	Endometrioid	G3	No
16	35	Yes	Yes	Yes	1-	1-	No	No	Clear cells	G3	No
17	59	Yes	No	No	2-	2-	No	No	Endometrioid	G2	No
18	62	Yes	Yes	No	1-	1-	No	No	Endometrioid	G1	No
19	85	Yes	No	No	1-	1-	No	No	Endometrioid	G2	Yes
20	49	Yes	Yes	No	2-	2-	No	No	Endometrioid	G3	Yes
21	58	Yes	Yes	No	1-	2-	No	No	Endometrioid	G2	No
22	60	No	Yes	No	No	No	3-	23-	Endometrioid	G2	Yes
23	56	Yes	No	No	3-	4-	No	No	Endometrioid	G1	No
24	57	Yes	No	No	2-, 1+	3-	No	No	Endometrioid	G1	No
25	87	Yes	No	No	2-	2-	No	No	Clear cells	G3	No
26	57	No	Yes	Yes	No	No	3-, 1++	2-, 1++, 1+	Endometrioid	G2	Yes
27	41	Yes	No	No	1-	2-	No	No	Endometrioid	G2	No
28	63	No	Yes	Yes	No	No	3-	3-	Serous	G3	Yes
29	70	Yes	No	No	2-	2-	No	No	Endometrioid	G1	Yes
30	60	No	Yes	No	No	No	7-, 1+	19-	Endometrioid	G2	No
31	57	No	Yes	Yes	No	No	7-	7-	Endometrioid	G1	No
32	60	Yes	Yes	No	1-, 1+	2-	No	No	Endometrioid	G3	No
33	63	No	No	No	No	No	2-	4-	Endometrioid	G2	No
34	56	No	Yes	No	No	No	3-	16-	Serous	G3	No
35	77	Yes	Yes	No	1-	1-	No	No	Endometrioid	G2	No
36	82	Yes	No	No	1-	1-	No	No	Endometrioid	G3	Yes
37	67	No	Yes	No	No	No	3-	7-	Endometrioid	G3	No
38	61	No	Yes	Yes	No	No	6-	6-	Endometrioid	G3	Yes
39	71	Yes	No	No	2-, 1+	3-	No	No	Serous	G3	No
40	79	Yes	Yes	No	1-	2-	No	No	Serous	G3	Yes

SLN: sentinel node; Pe: pelvic; Ao: aortic; LFN: systematic lymphadenectomy; FS: frozen section analysis: LVSI: lymph-vascular space involvement; -: negative lymph node; +: micro-metastasis; ++: macro-metastasis.

Overall 110 lymph nodes were analyzed by OSNA and frozen section analysis, and 8 (7.3%) positive nodes were found: micro and macro-metastases were detected in 7 and in 1 case, respectively.

In a single case, both OSNA assay and frozen section did not detect any metastasis in the SLN, whereas the final histopathological examination was positive for micro-metastasis in 1 non-SLN.

Using the breast cancer cut-off value to find lymph node metastasis [14] and considering together micro and macro-metastases, OSNA assay sensitivity and specificity were 87.5% and 100% respectively. Positive and negative predictive values were 100% and 99% respectively, with an accuracy of 99%. As far as frozen section examination and subsequent ultra-staging analysis are concerned, we reported sensitivity and specificity of 50% and 94.4% respectively; positive and negative predictive values were 14.3% and 99%, respectively, with an accuracy of 93.6%. We found 7 discordant diagnoses (6.4%) in 7 different patients. Specifically, in a single case (2.5%) OSNA assay was negative, while frozen section/ultra-staging examination found a micro-metastasis. In six cases (15%) OSNA assay resulted positive while frozen section/ultra-staging examination was negative: in 1 (2.5%) case the level of CK19 mRNA was higher (1700 copies), and in the remaining was < 500 copies.

## Discussion

Sentinel lymph node biopsy has been recently introduced in the staging procedures of early-stage endometrial cancer patients: it reduces the intraoperative as well as the long-term morbidity of a systematic lymphadenectomy [23], and, thanks to the ultra-staging analysis, improves the detection rate of the micro-metastasis and the isolated tumor cells [12, 24]. With the aim to reduce the rate of false negative cases, a surgical algorithm retroperitoneal staging was firstly proposed by the Memorial Sloan Kettering Cancer Center: the observance to that protocol, by performing systematic lymphadenectomy when SLN is/are not mapped, allows reducing the false negative rate of the SLN mapping [25]. This innovative approach to nodal mapping has been recently included in the NCCN guidelines for low- and intermediate-risk stage I endometrial cancer patients [9]. More recently, SLN mapping was considered also for high-risk and type II endometrial cancer patients, showing survival outcomes superimposable to those reported for systematic lymphadenectomy [26–27].

A hypothetical limit of this approach is the absence of intra-operative diagnosis of nodal status and its potential indication to perform systematic lymphadenectomy in presence of SLN nodal metastases. In fact, the presence of micro- and macro-metastases represents not only an indication for adjuvant therapy but also a risk factor for non-SLNs metastases. To date, patients with diagnoses of micro- or macro-metastases at ultra-staging were treated with adjuvant chemotherapy and radiotherapy. Theoretically a systematic pelvic and aortic lymphadenectomy in presence of metastatic SLN could avoid the need of adjuvant radiotherapy limiting the risk of treatment complication improving the patients’ quality of life.

OSNA assay has been proposed as an effective tool for intra-operative detection of nodal micro and macro-metastases in different solid tumors [28–30]. In the present study 110 lymph nodes from 40 FIGO stage I EC patients were intra-operatively analyzed by both OSNA and frozen section examination.

At definitive pathology, the residual LN tissue was processed with step-level sections (each step at 200 micron) including haematoxylin and eosin (H&E) and IHC slides, then examined using a light microscope. In contrast to other known protocol of ultra-staging analysis [12], our in house protocol consisted in step-level sections, with each step at 200 micron (and not at 50 micron), up to consume the all tissue of the paraffin-block with at least ten slides per block (and not only a total of five slides per block).

In the present study, using the validated OSNA diagnostic criteria for breast cancer [14, 22], sensitivity, specificity and overall diagnostic accuracy were 87.5%, 100% and 99% respectively. We found that OSNA analysis is very sensitive and specific for the identification of nodal metastases in EC patients, and are superimposable to those recently reported by López-Ruiz et al. [20].

Overall, we found 8 nodal metastases and a total of 7 discordant diagnoses in the 7 corresponding patients. In all discordant cases, a micro-metastasis was found: in 6 patients we found positive OSNA assay with negative frozen section/ultra-staging analysis, and in the remaining case negative OSNA assay and positive histopatological analysis.

An accurate intra-operative technique for nodal diagnosis could be considered a relevant tool in the tailoring of nodal staging.

Differently from those previously reported by Lopez-Ruiz et al. [20], we did not found true false-positive cases due to the presence of benign glandular epithelial inclusions. Despite the very low rate of this finding reported in the literature [31–32], false-positive nodes caused by benign glandular epithelial inclusions, mainly associated with previous pregnancies [20], can be a tricky issue since it does not allow morphological correlation with a positive result at the OSNA assay. Nevertheless, according to other papers performing molecular technique probing for cytokeratins, we consider that a reasonable incidence of false positive cases of approximately 0.2% in pelvic and aortic lymph nodes does not invalidate the method [20].

We could suppose that all the discordant diagnoses are due to the fact that micro-metastasis might have been present only in a portion of tissue cut and sent for only one of the two methods. It is clear in fact that the tissue sent for OSNA assay cannot be use for histology, and conversely the formalin-fixed and paraffin-embedded tissue in not suitable for molecular analysis. Different sample preparation methods have been proposed in order to reduce the risk of this unavoidable bias in a validation and feasibility study [20]. Nevertheless, validation studies of OSNA assay in EC could recommend it as a useful tool for the intraoperative diagnosis of SLN metastases, as it has been also reported in breast cancer [28].

Despite the limit of this study, mainly represented by the small sample size and the low number of metastatic nodes, we confirmed that the OSNA assay measuring CK19 mRNA copy numbers could be used as a novel tool for the analysis of SLN in early stage EC patients. We can conclude that the combination of OSNA procedure with the sentinel lymph node mapping could represents an efficient an intra-operative tool for the selection of early-stage EC patients to be submitted to systematic lymphadenectomy. Waiting for the results of the ENDO-OSNA prospective multicenter validation study, further studies should define the prognostic impact of molecular assessment of SLN and the non-SLNs management in EC patients.
